# Increasing exposure to potential flooding in high-mountain catchments

**DOI:** 10.1038/s41598-026-70268-0

**Published:** 2026-10-08

**Authors:** C. Scott Watson, Lauren D. Rawlins, Mohan B. Chand, Rakesh Bhambri, Nitesh Khadka, Milan Shrestha, Deepak KC, Daniel Routledge, Grace Middleditch

**Affiliations:** 1 School of Geography and water@leeds, University of Leeds, Leeds, LS2 9JT UK; 2 Department of Environmental Science, Himalayan Cryosphere, Climate and Disaster Research Center (HiCCDRC), School of Science, Kathmandu University, Dhulikhel, Kavre Nepal; 3 Department of Geography, University of Delhi, New Delhi, India; 4 Centre for Water Resources Studies (CWRS), Institute of Engineering, Tribhuvan University, Kathmandu, Nepal; 5 School of Sustainability, Arizona State University, Tempe, Arizona USA; 6 United Nations Development Programme Nepal, Kathmandu, Nepal; 7 Institute of Earth Sciences, University of Lausanne, Lausanne, Switzerland

**Keywords:** Climate sciences, Environmental sciences, Hydrology, Natural hazards, Water resources

## Abstract

Exposure to flood hazards in high mountain catchments is expected to increase alongside natural hazards, driven by climate change, glacier retreat, and landscape evolution, yet temporally consistent, high-resolution exposure data remain scarce. Here we quantify changes in the exposure of buildings and people to flooding in Nepal and transboundary regions of India and China. Validation against a manually derived reference dataset revealed considerable differences in accuracy and completeness across five building footprint products. Google Open Buildings Temporal 2.5D (OBT) displayed the highest median accuracy (F1 = 0.68), followed by Google Open Buildings V3 (F1 = 0.62). We assessed exposure relative to river channels using the height above nearest drainage (HAND) metric and modelled glacial lake outburst flood (GLOF) inundation extents. Building area within 0–50 m vertical proximity to channels increased by 46% between 2016 and 2023, while those located within modelled GLOF extents increased by 77%. Population projections indicate continued growth in areas of potential flood exposure, with 18% (*n* = 293,800) of the study-area catchment population estimated to reside within 0–50 m HAND in 2026. Our results demonstrate rapidly increasing exposure in Himalayan catchments and highlight the importance of validated, multi-temporal exposure datasets to inform disaster risk reduction.

**Supplementary Information:**

The online version contains supplementary material available at https://doi.org/10.1038/s41598-026-70268-0.

## Introduction

Flood hazards in high-mountain catchments, such as the Himalaya, are a function of seasonal monsoonal dynamics^1,2^, extreme precipitation events^3,4^, and rare but high-magnitude debris flows and glacial lake outburst floods (GLOFs)^5^. Steep topography and strong altitudinal climate gradients mean that even small changes in temperature or precipitation can produce disproportionate impacts on the cryosphere and downstream hydro‑ecological systems^6,7^. A warming landscape with increased precipitation extremes is preconditioned for an increased frequency of flooding and mass movement events^8,9^, that can cause secondary flooding, or transition into damaging debris flows^10^. Similarly, widespread glacier mass loss is increasing the number of glacial lakes^11–13^ and therefore potential GLOF occurrence^14–17^. This is coupled with high potential socio-economic impacts of floods in Himalayan countries such as Nepal, India, China, and Bhutan^18–21^, and the occurrence of recent damaging flood events^22,23^. Additionally, the exposure of people, buildings, and infrastructure in high-mountain catchments is expected to be increasing^24^, coincident with population growth^25^, migration to lower floodplains^26^, increased tourism and recreation^27–30^, and growing economic activities including hydropower development that is targeted to capture snow and glacier meltwater runoff^31,32^. However, studies assessing extreme floods often focus on the hazard, rather than exposure or vulnerability^33^. Exposure refers to the presence of people, infrastructure, and other elements in places where they could be affected by a hazard, whereas vulnerability reflects the capability and adaptive capacity of exposed elements to respond to a hazardous event, determined by socio-economic factors such as income level, land tenure, and social and human capitals, which can influence whether or not individuals and social groups are at-risk^34,35^. Quantifying dynamic exposure to increasingly prevalent natural hazards including flooding and mass movement events is a high priority^36–38^. Global exposure to GLOFs has previously been estimated by applying a 1 km wide horizontal buffer to river channels downstream of glacial lakes and summing the population within this zone, providing a first order approximation of potential impacts^39^. However, this approach does not account for vertical proximity to flow paths, which changes rapidly in steep mountain catchments. In contrast, the height above nearest drainage (HAND) metric defines a vertical buffer relative to channel elevation, to quantify exposure within a given vertical height of the channel. This offers an alternative method of calculating relative flood exposure, that similarly does not require a probabilistic flood return period framework^40–43^.

Risk assessments require spatially and temporally consistent exposure datasets, including buildings, roads, and population estimates. Yet even fundamental data such as building footprints are known to contain notable completeness issues and spatial biases for both highly urbanised and rural regions^44–46^. Data completeness is particularly challenging in mountain catchments, owing to the lower availability of high-resolution satellite imagery that is used to derive building footprints. Similarly, the recent prevalence of using deep learning methods to automatically extract building footprints^47–49^ could be similarly biased by persistent cloud cover and lower availability of training data in mountain catchments. Therefore, time-consuming manual checking and updating of existing inventories is often required before quantifying flood impacts^19,50^. Additionally, global building inventories are often provided without a clear timestamp, as many are derived from composites of high-resolution satellite imagery spanning multiple years^48^. Although global urban growth products derived from long-term Landsat image archives are now available^51^, it is only with recent developments of deep learning models that building footprints can be derived using the more recent and higher resolution archive of 10 m resolution Sentinel-2 data, for example Google’s Open Buildings Temporal 2.5D dataset^49^. However, evaluations of these datasets are lacking, particularly for topographically complex mountain catchments where imagery is sparse and where orthorectification errors can introduce positional shifts in derived datasets^52,53^.

This study aims to reveal how and where the exposure to potential flooding is changing in high mountain catchments of Nepal and transboundary regions of India and China. Our specific objectives are: (1) to assess the completeness and accuracy of open access building footprint datasets in topographically complex mountain catchments, (2) use this evaluation to produce a building footprint exposure dataset that is consistent in space and time, and (3) to quantify recent changes in the exposure of buildings and populations to potential flooding, and discuss the implications for disaster risk management, with particular attention to extreme events such as GLOFs.

**Fig. 1:**
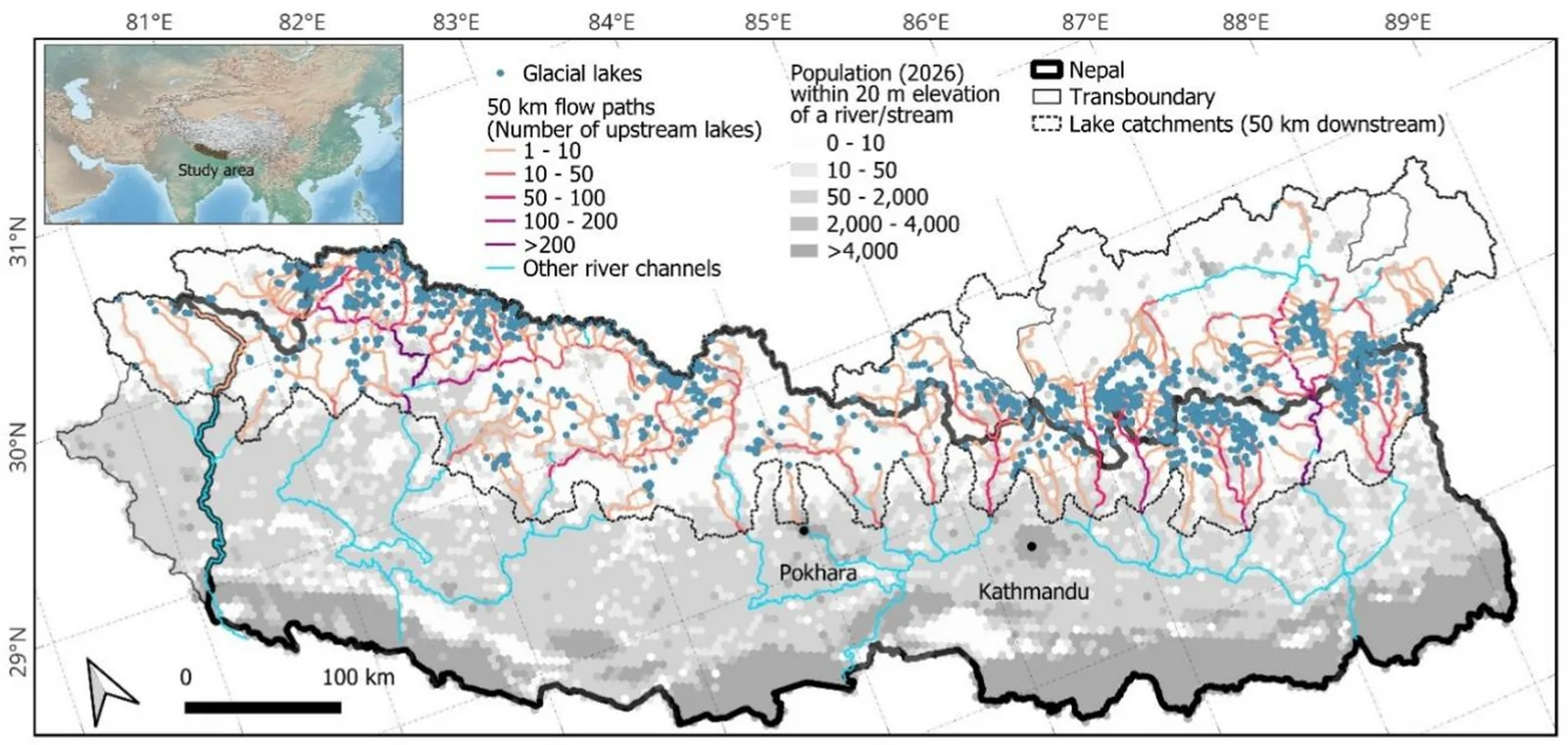
Map of Nepal and transboundary areas in India and China (Tibet). Moraine-dammed glacial lakes^54,55^ are shown with 50 km downstream flow paths, which are coloured by the number of upstream glacial lakes. The catchments of these flow paths form the main study area. Populated cells are shown from 2026 WorldPop data^56^ for areas within 20 vertical meters of a water course. Only major water courses are shown on the map. Figure created in QGIS 3.34.14.

## Results

In the Nepal and transboundary region (Fig. 1), the number of buildings between datasets ranged from 6,097,415 (Google Open Buildings Temporal 2.5D (OBT) to 14,496,826 (OpenBuildingMap), and the area of buildings ranged from 628 km^2^ (OpenStreetMap) to 1,117 km^2^ (OBT) (Table 1). Therefore, OBT featured the largest building area coverage, but the smallest number of individual building polygons. OBT buildings in the glacial lake catchments (dashed line Fig. 1) (Table 2) represented 9% (number) and 7% (area) of the buildings in the full Nepal and transboundary region (Table 1). For example, OBT featured 528,025 buildings covering 75.19 km^2^ of the glacial lake catchments. Buildings in Nepal and India were generally distributed at lower elevations (< ~ 4,000 m asl) compared to those in China, which were situated on the Tibetan Plateau (Supplementary Fig. 1a–c). Additionally, buildings in China were vertically closer to glacial lake flow paths (smaller HAND values) with ~ 70% of building area within 100 m vertically of the flow paths, compared to those in Nepal and India with ~ 15–20% within 100 m (Supplementary Fig. 1d–f).

**Fig. 2:**
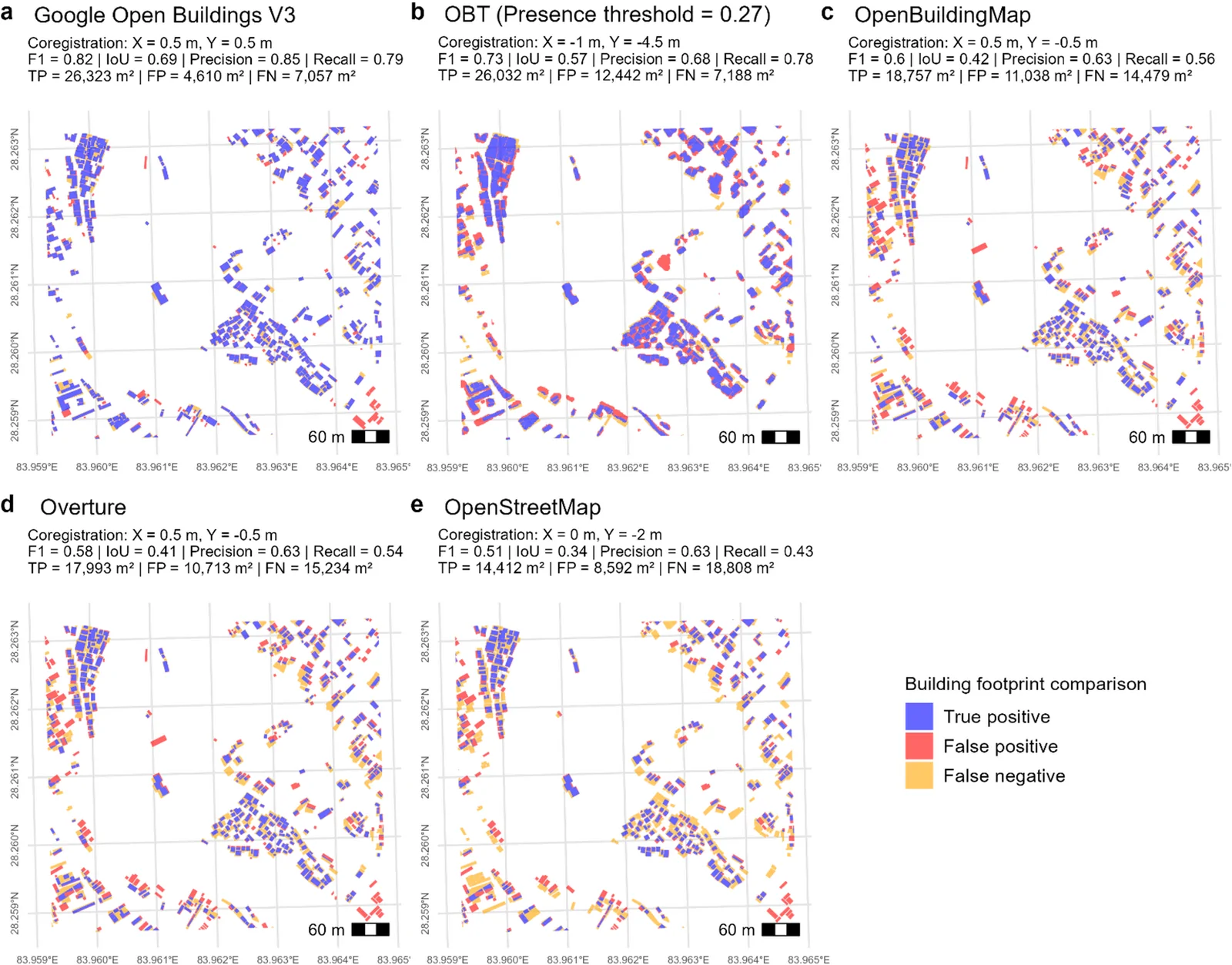
Example comparison of building footprint datasets for an area of Lamachur on the Seti River near the city of Pokhara, Nepal. Building footprints were compared to a manually derived reference dataset for 2023. Coregistration values represent a horizontal (x, y) shift applied to the footprints to best align with the reference data. Accuracy assessment metrics are also shown including F1 score, intersection over union (IoU), precision, recall, true positives, false positives, and false negatives. Figure created in R ggplot2 v3.5.2^57^.

Open access building footprint datasets were compared with manually derived reference data to estimate their completeness and area accuracy (e.g. Fig. 2). Across 13 validation sites and 5,994 reference buildings, OBT displayed the highest median F1 score (0.68), followed by Google Open Buildings V3 (0.62) (Supplementary Fig. 2a), whereas OpenBuildingMap, OpenStreetMap, and Overture each featured a median F1 score of 0.51. Where satellite imagery was available, multiple dates of building footprints were digitised at each validation site since the date of the open access building datasets was not known, with the exception of OBT. Therefore, the comparisons represent an indicative accuracy assessment but were not a like-for-like comparison of building polygons extracted for the same date. OBT required the largest horizontal coregistration shift (3.1 m) to align with the reference data, whereas Google Open Buildings V3 featured the lowest (1.58 m) (Supplementary Fig. 2b). However, the reference data did not necessarily have the best absolute positioning, since this could not be evaluated across the full study domain. Therefore, we did not shift the OBT data before the regional exposure assessment.

**Table 1:** Building footprint count and area for the Nepal-transboundary region.

Dataset	Nepal-transboundary		Nepal	
	Count	Area (km^2^)	Count	Area (km^2^)
Google Open Buildings V3	12,421,041	736.06	11,688,517	691.20
OBT^1^	6,097,415	1117.37	5,796,908	1058.33
OpenBuildingMap	14,496,826	802.08	13,767,537	755.78
Overture	13,341,790	757.65	12,908,963	724.52
OpenStreetMap	8,105,059	628.12	8,057,090	622.39

^1^Calculated using a presence threshold of 0.27 for year 2023 and filtered using a snow and elevation mask (see methods).

**Table 2:** Building footprint count and area for the Nepal-transboundary region and respective countries within the 50 km downstream catchments.

Dataset	Nepal-transboundary		Nepal		China		India	
	Count	Area (km^2^)	Count	Area (km^2^)	Count	Area (km^2^)	Count	Area (km^2^)
Google Open Buildings V3	640,681	37.53	580,955	33.57	13,486	1.47	46,240	2.49
OBT^1^	528,025	75.19	468,146	60.85	49,098	12.64	10,781	1.71
OpenBuildingMap	911,315	49.65	841,308	43.48	22,961	3.62	47,046	2.55
Overture	836,084	47.23	798,067	41.99	23,207	4.14	14,810	1.10
OpenStreetMap	534,228	32.78	516,443	29.46	14,225	3.03	3,560	0.29

^1^Calculated using a presence threshold of 0.27 for year 2023 and filtered using a snow and elevation mask (see methods).

**Fig. 3:**
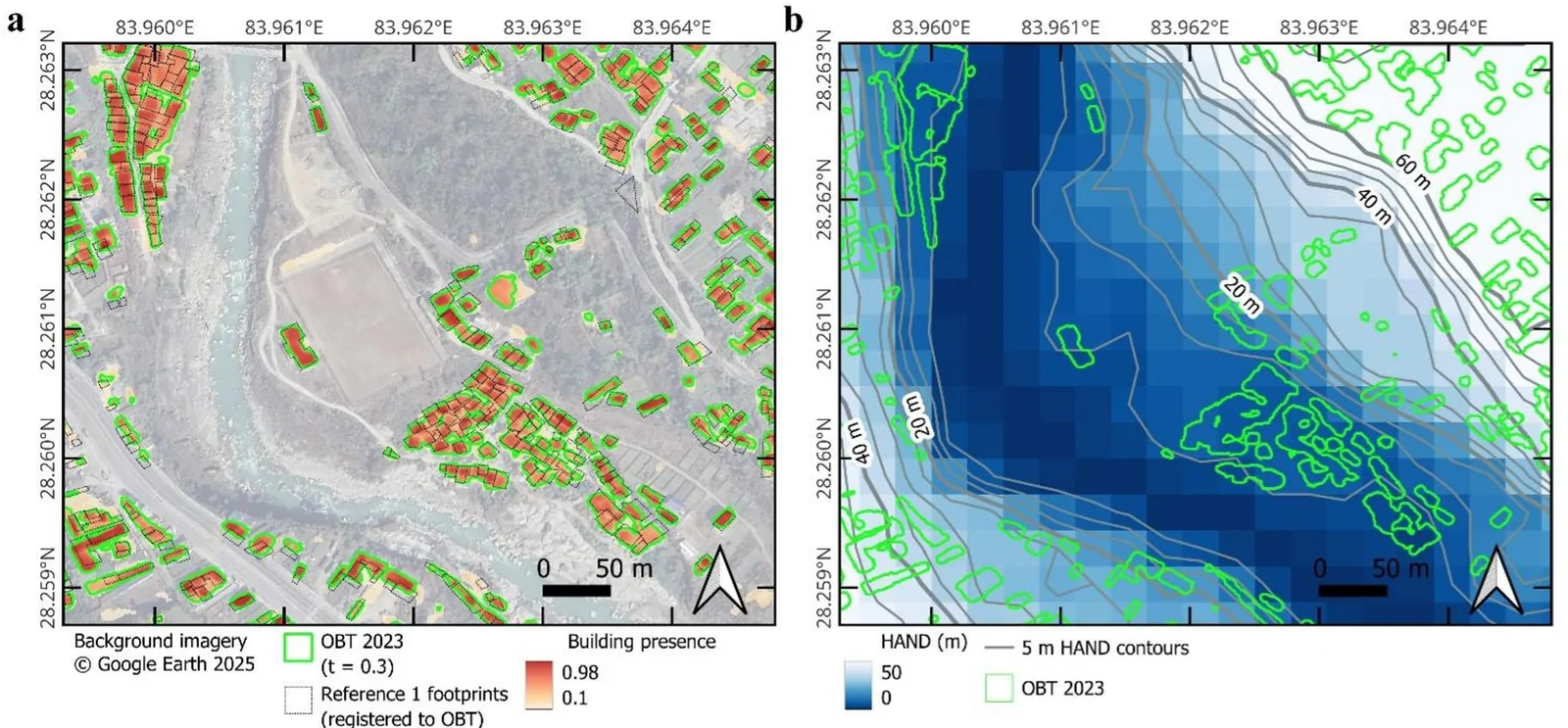
Example building footprint datasets at Lamachur on the Seti River near the city of Pokhara, Nepal. (**a**) Comparison of the Open Buildings 2.5D Temporal (OBT) dataset with a manually derived reference building footprint dataset for year 2023. Building outlines derived from thresholding the OBT data (t = 0.3) are shown, along with the reference footprints. (**b**) HAND data and respective HAND contour lines for the same area as (**a**), shown with OBT footprints. Figure created in QGIS 3.40.11.

**Fig. 4:**
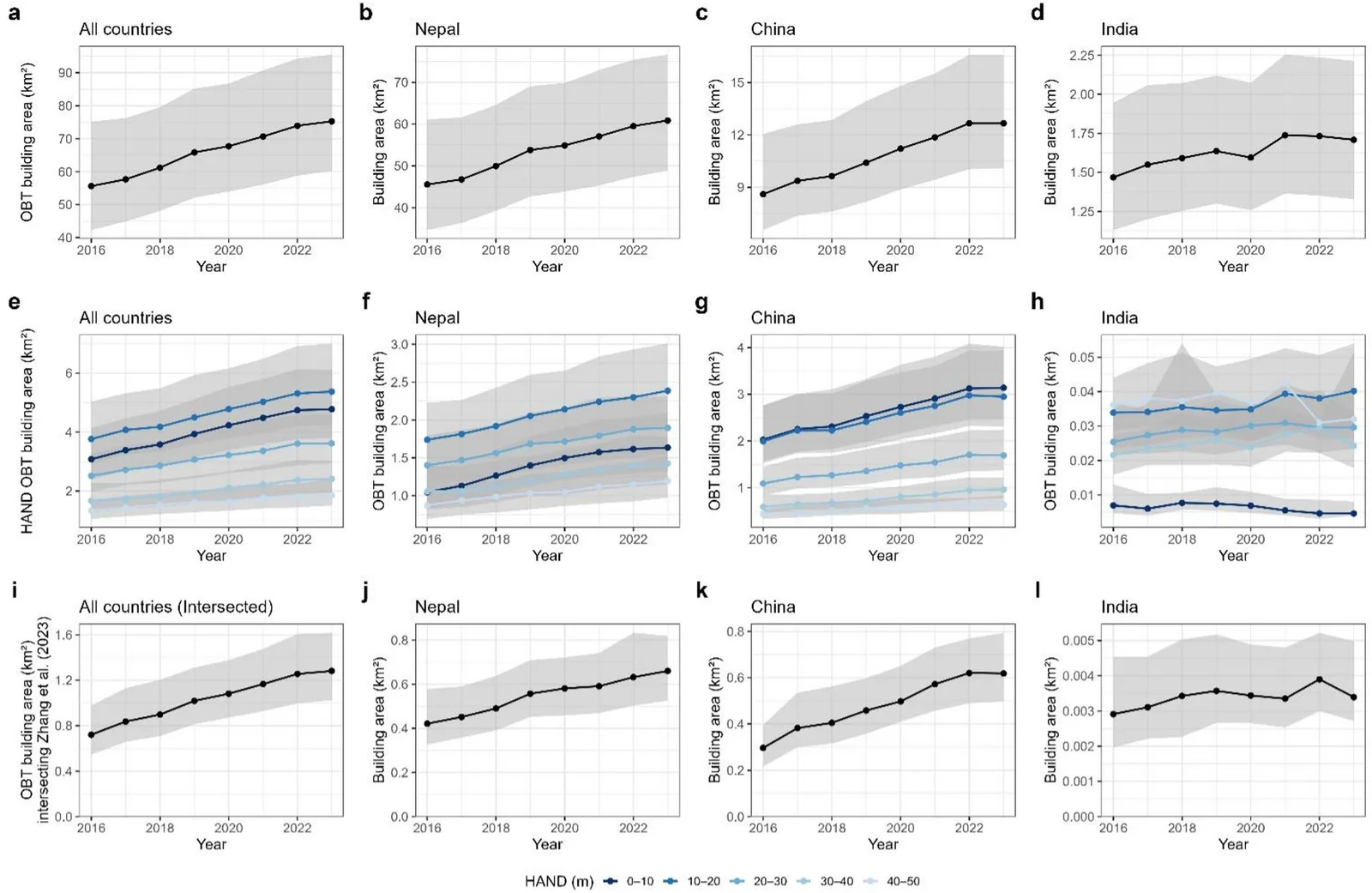
Change in building area through time in the glacial lake catchments, plotted by transboundary catchment countries. (**a**–**d**) Total building area per year from the OBT dataset shown for an extraction threshold of 0.27 (black line) and confidence interval (grey ribbon) using thresholds of 0.18 to 0.37 (see methods). (**e**–**h**) Building area intersecting with increasing HAND bins from 0 to 50 m above the nearest river channel. (**i**–**l**) Building area intersecting with the flood extents of Zhang et al. (2023).

OBT building footprints were derived from a raster-based confidence metric, which produces less regularised boundaries and distinction between neighbouring footprints (e.g. Fig. 3a). Nonetheless, since OBT was the only multi-temporal dataset and featured the highest F1 score, it was used to evaluate changing exposure in flood-prone areas derived from HAND (e.g. Fig, 3b), and by intersecting with the GLOF flow paths of Zhang et al. (2023). Across all regions, the area of buildings in glacial lake catchments increased through time (2016–2023) (Fig. 4a), which was a similar trend for buildings vertically closest to the river channel, except for India where there was no clear change (Fig. 4e–h). The total area of buildings closest to the river channel (HAND 0–10 and 10–20 m) was generally larger than those situated higher (Fig. 4e–h). For buildings within 0–50 m HAND, area increased from 12.38 km^2^ (OBT threshold range 9.65–16.42 km^2^) to 18.04 km^2^ (OBT threshold range 14.44–23.16 km^2^), or a 46%±5% increase between 2016 and 2023 (Fig. 4e). Here the OBT threshold range represents the median threshold value (0.27) and 5th and 95th percentiles (0.18 and 0.37) that were used to produce three classifications of the OBT data to define uncertainty bounds (see Methods). Building area within the modelled GLOF extents of Zhang et al. (2023) similarly increased through time, although again, this was less apparent for buildings in India (Fig. 4i–l). For buildings within the GLOF inundation extents of Zhang et al. (2023), area increased 0.72 km^2^ to 1.28 km^2^, or a 78 ± 13% increase between 2016 and 2023 (Fig. 4i).

**Fig. 5:**
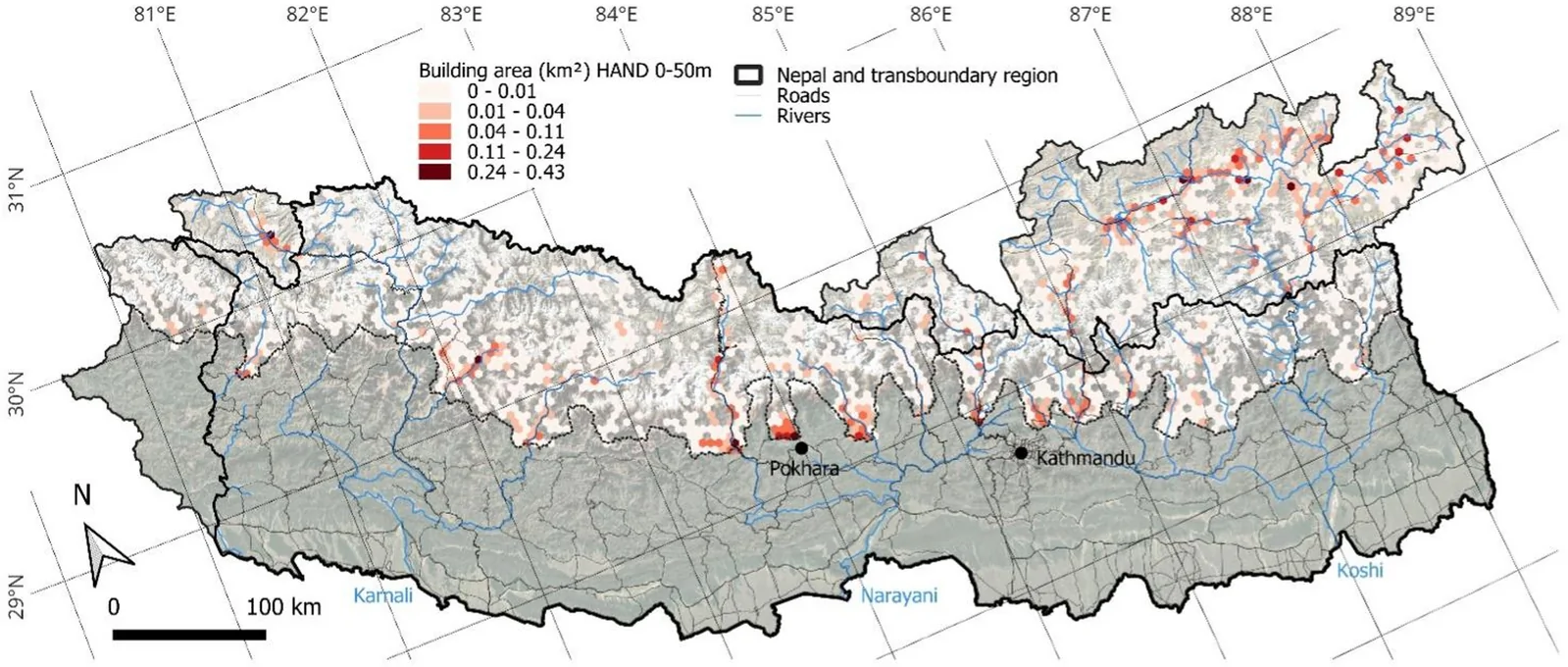
Building exposure hotspots from OBT 2023 data summed to ~ 6 km diameter cells. Figure created in QGIS 3.40.11.

Hot spots of building exposure were distributed across the study but were less pronounced in the northeast and northwest of Nepal (Fig. 5). In Nepal, exposure was concentrated in the lower reaches of the catchments, whereas in the transboundary region of the Koshi river catchment in China it was more evenly distributed, with an approximately equal proportion located 0–25 km and 25–50 km downstream (Fig. 5; Supplementary Fig. 3).

**Fig. 6:**
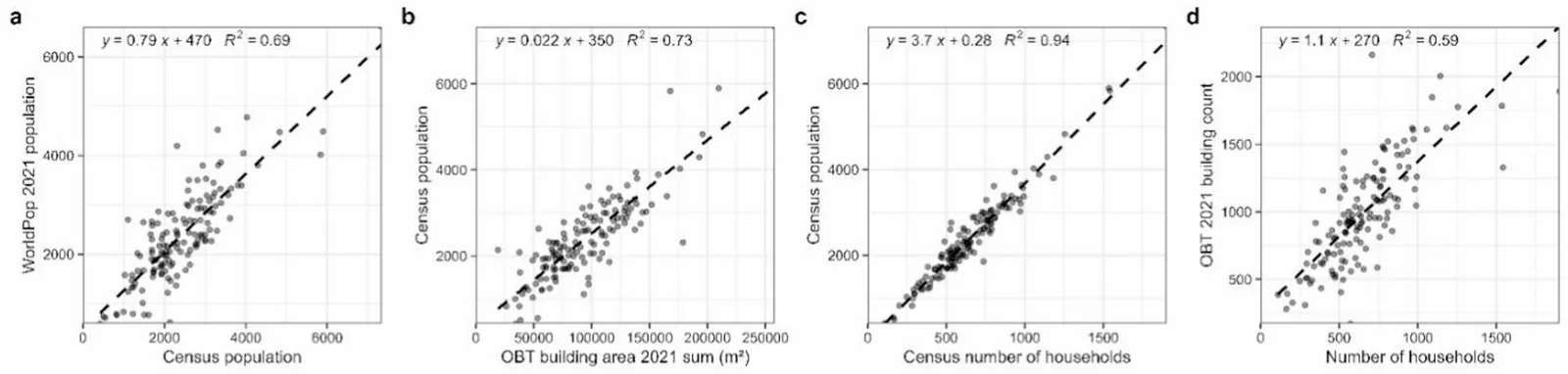
Linear regressions of Nepal 2021 census data with WorldPop 2021 and OBT building footprints for 140 wards. (**a**) Census population and the WorldPop population summed within the census ward boundaries. (**b**) OBT 2021 building area summed within the ward boundaries and the census population. (**c**) Number of households and the population summed by ward. (**d**) Number of households and the OBT 2021 building count summed by ward.

**Fig. 7:**
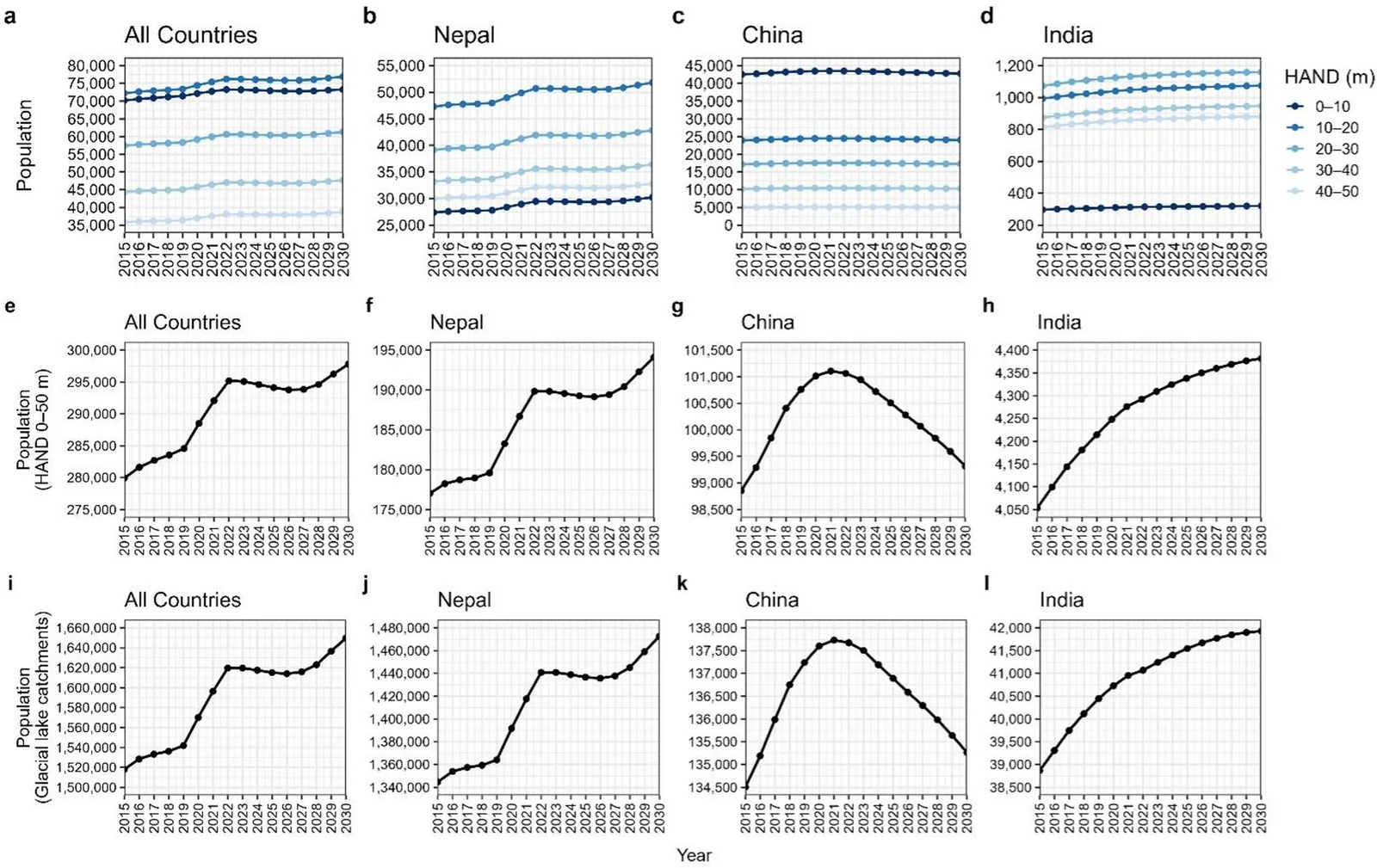
Change in population through time from WorldPop data in the study catchments. (**a**–**d**) Population summed by HAND bin from 0 to 50 m above the river channel. (**e**–**h**) Population summed into a single HAND bin of 0 to 50 m above the river channel. (**i**–**l**) Total population in the lake catchments.

Nepal census data correlated well (R^2^ = 0.69) with WorldPop data (Fig. 6). Similarly, OBT building area correlated well with population (R^2^ = 0.73), which reflected a linear trend between the census-measured number of households and total population (R^2^ = 0.94) (Fig. 7). The correlation between the number of households reported in the census and OBT building footprints was weaker (R^2^ = 0.59), likely reflecting the less regularised footprints of OBT and lower count compared to other datasets as described earlier. Since the census data did not cover the full study region and was for a single point in time, WorldPop data was used to quantify changing population exposure. However, since we only compared the WorldPop data to a subset of census data for one year (2021) and only for Nepal, there is an unknown uncertainty in the future WorldPop projections. Population was projected to increase through time in all study locations other than catchments in China, where there was a downward trend from 2021 (Fig. 7).

Population change was evaluated alongside HAND as a proxy for potential flood exposure using WorldPop data projections. Changes were quantified separately using (1) HAND for all waterways in the glacial lake catchments, which represents overall potential flood exposure, and (2) HAND specifically for glacial lake flow paths, which represents populations that could be affected by GLOFs. Across the study catchments, population closest to the river channels (HAND 0–10 m) increased from 70,200 to 73,273 (2015–2030), or a 4% increase (Fig. 7a). Specifically for glacial lake flow paths, the increase was 7% (30,300 to 32,500, 2015–2030) (Supplementary Fig. 4a). Similarly, population within 0–50 m HAND was projected to increase by 17,900, 2015–2030 (6% increase) (Fig. 7e), and 10,300 (8% increase) for glacial lake flow paths (Supplementary Fig. 4b). The projected 2026 population within 0–50 m HAND (293,800) represented 18% of the total population in the glacial lake catchments (1,613,900) (Fig. 7e, i), or 9% (144,400) for glacial lake flow paths (Supplementary Fig. 4e). For glacial lake flow paths in Nepal, there were an estimated 108,500 people within 0–50 m HAND in 2026 (Supplementary Fig. 4f), of which 22,800 were within 0–10 m HAND (Supplementary Fig. 4b).

## Discussion

In a warming climate, high‑mountain catchments are increasingly preconditioned for flooding as intensified precipitation extremes interact with glacier retreat, emerging glacial lakes, permafrost degradation, and landscape instabilities that amplify flood hazards^58,59^. Reducing this risk requires spatially and temporally consistent and up-to-date exposure data that is often lacking in these areas. Our study found substantial variation in both the number and total area of buildings captured across five widely used open-access building footprint datasets for Nepal and its transboundary region, with direct implications for accurately estimating exposure to natural hazards (Tables 1 and 2). Building counts ranged from 528,025 (OBT) to 911,315 (OpenBuildingMap), while areas ranged from 32.8 km² (OpenStreetMap) to 75.2 km² (OBT) (Table 2). OBT featured the highest median accuracy (F1 = 0.68) and served as the only time-stamped and multi-temporal exposure product (2016–2023). The moderate F1 score of OBT (0.68) illustrates that even the best-performing dataset contains notable omission and commission errors, highlighting the uncertainty that building footprint products can introduce into regional-scale exposure analyses. Google Open Buildings V3 followed closely (F1 = 0.62), whereas OpenBuildingMap, Overture, and OpenStreetMap each produced median F1 scores of 0.51. OpenBuildingMap and Overture merged OpenStreetMap data with other machine-learning derived datasets, which accounts for their similar accuracy assessment scores, and suggests that the machine-learning data did not notably improve on OpenStreetMap’s heterogenous completeness^44,45,60^. However, the lower building counts and less regularised building polygons in OBT also highlights the trade-off between the semantic accuracy of individual building polygons, the geometric detail of the polygons, and their temporal consistency^49,61^.

### Spatial and temporal exposure trends

Building exposure within the glacial lake catchments represented only 6% of the total Nepal-transboundary building area, and patterns varied between subcatchments in Nepal, China, and India. In China (Tibetan Plateau), buildings tended to occur at higher elevations but were vertically closer to flow paths, with ~ 44% of OBT building area lying within 50 m HAND. In contrast, buildings in Nepal and India were generally confined to narrower, and high-relief corridors at lower elevations with only 4% (India) and 10% (Nepal) within the same vertical buffer (Supplementary Fig. 1). This topographic influence on settlement patterns reflects broader, flatter plateau landscapes of the Tibetan Plateau that permit closer riverine development, compared to the steep-sided valleys typical of Nepal and India. This also demonstrates the value of the HAND metric in evaluating relative exposure using vertical position above the river channels, whereas reliance on planimetric horizontal distance buffers from a river channel would overestimate flood exposure, since elevation increases rapidly with horizontal distance^39^. Interactions between valley confinement, infrastructure development, and flood impacts have been documented following recent Himalayan flood disasters, including the Melamchi (2021) and Sikkim (2023) events, where channel erosion and sediment mobilisation through cascading geomorphic processes, amplified downstream impacts beyond direct inundation alone^22,62,63^.

The observed 46% increase in building area within 0–50 m HAND, together with a 77% increase in building area intersecting published GLOF inundation extents between 2016 and 2023 (Fig. 4e), indicates rapid encroachment into areas of potential flood exposure. These increases reflect the expansion of infrastructure, tourism facilities^29,64^, and hydropower^31,65^, combined with limited implementation of land-use plans^66^. In Nepal, this reflects a strategic policy for risk sensitive land use planning based on multi-hazard risk assessments^67^, but without regulatory implementation. Therefore, infrastructure development into river corridors and low-lying floodplains restricts natural flow pathways and flood water storage^68,69^. Additionally, unregulated tourism-driven encroachment along river corridors has been documented in many Indian Himalayan valleys (e.g., Dharali)^70^. Increases in building exposure through time were evident across Nepal and China but showed no clear trend in the transboundary catchments of India, likely due to a combination of factors. First, the Indian portion represents a comparatively small fraction of the overall study area, resulting in far fewer buildings (∼10,000–47,000 versus hundreds of thousands in Nepal). Second, Nepal has experienced prominent informal settlement expansion and tourism-driven construction along some river corridors (e.g., Lamachaur near Pokhara) (Fig. 5)^71,72^. In contrast, the Indian transboundary zones may be subject to stricter border-region regulations and more rigorous environmental-impact assessments^73^, lower economic incentives for valley-floor development, or greater out-migration pressures^74^. Further targeted, finer-scale analyses are necessary to link socio-economic or policy drivers with the observed trends.

### Population trends

National census data indicate declining populations in some mountainous wards in Nepal due to out-migration^75^, highlighting a potential divergence in trends between structural exposure and population. In contrast, WorldPop trends suggest continued population growth, other than in the sub-catchment spanning China (Fig. 7). However, these projections carry uncertainty, particularly in rural and mountainous settings where gridded models may underestimate dispersed populations^76^. Nonetheless, the continued expansion of infrastructure and assets such as hydropower are often accompanied by population growth^77^, and are often concentrated within flood hazard zones, particularly along riverbanks and the valley bottoms. For instance, the Timure inland port in Nepal along China-Nepal economic corridor built on the riverbanks was destroyed by 2025 transboundary GLOF from China^78^. The roadway connecting Nepal and China through the Himalayas has expanded to six routes, primarily following river courses, while trading ports and settlements have also increased^79^. In the Kanchenjunga Conservation Area of eastern Nepal, there has been a dramatic rise in tourist lodges and road networks over the past 25 years^64^, which has followed similar trends in Sagarmatha National Park^80^. At a broader scale, national-level assessments indicate that unplanned urban growth and infrastructure expansion along river corridors has generated new forms of exposure to climate-related hazards, with a rising number of people and assets affected by flooding in recent years^62,81,82^. This demonstrates a compounding risk dynamic in which intensifying hydroclimatic extremes interact with riparian development trajectories to amplify flood risk to settlements and assets, such as hydropower and transport infrastructure.

For glacial lake flow paths in Nepal (Fig. 1), there were an estimated 108,500 people exposed to potential flooding in 2026 (within 0–50 m HAND) (Supplementary Fig. 4f). Direct comparison with previous studies was not possible because other assessments using similar methods were unavailable. Taylor et al.^39^ found approximately 832,000 people in glacial lake flow paths in Nepal in 2020. This estimate is not directly comparable to our results because the study used different datasets and exposure modelling approaches, including a 1 km horizontal buffer around river channels to delineate areas of potential flood exposure and a different population dataset (2020 Gridded Population of the World Version 4).

### Limitations and future work

Exposure of people to natural hazards and its temporal trends are inherently dynamic and are often conditioned by a complex set of socio-economic factors that determines varied levels of exposure and vulnerability to these hazards. Population distributions and building occupancy can change rapidly in response to extreme events (e.g. earthquakes or pandemics)^83^, longer-term outmigration driven by education and employment opportunities^84,85^, and daily mobility patterns that determine whether people are at home, work, or school at the time of a flood^86^. These dynamics are not captured in annually aggregated building infrastructure or population exposure estimates. Methodological limitations also influence the exposure estimates. For example, population exposure was estimated using WorldPop data that were validated against census data^75^ for a single time point and multitemporal validation was not possible, which introduces uncertainty into projected population trends. However, OBT building area correlated strongly with population estimates (R² = 0.73), presenting its utility as a multi-temporal proxy where census data are incomplete or unavailable. Gridded population datasets such as WorldPop are also known to contain biases that underrepresent rural populations^76^. Additionally, we did not account for the vertical structure of buildings, although such datasets, often derived by deep learning approaches, are becoming available but are not widely validated^46,49,87^. HAND based proximity metrics are also linked to DEM resolution and accuracy, which increases uncertainty in steep terrain and may result in some buildings near class boundaries being assigned to adjacent HAND bins. However, since HAND represents a relative elevation difference referenced to the local drainage network, uncertainties are expected to be smaller than the absolute DEM elevation error. Although other global open access DEMs could also be evaluated for deriving HAND, the greatest improvements would come from the availability of higher resolution topographic data that are better able to resolve steep mountain topography.

Future work should extend the multitemporal validation of population and building footprint datasets while incorporating vulnerability indicators and flood hazard models that capture flood depth, velocity, and return period characteristics. Combined with exposure data, these components are necessary to quantify flood risk, since greater exposure does not directly equate to higher potential losses. Multitemporal exposure estimates can inform adaptation investments, early warning systems, and risk-informed planning at national and transboundary catchment scales that target exposure hot spots. Additionally, linking exposure datasets with early warning systems and operational monitoring enables impact-based forecasting, helping to shift disaster management from retrospective response to anticipatory and risk-informed action.

## Conclusions

Overall, our findings highlight rapidly increasing exposure to potential fluvial and GLOF related flooding in Himalayan catchments and underscore the importance of validating open datasets before their application in risk assessments. Comparison of five widely used building footprint inventories revealed substantial discrepancies in building counts and total area, with direct implications for quantifying exposure. Google Open Buildings Temporal 2.5D (OBT) featured the highest overall accuracy and was the only dataset supporting multitemporal analyses. However, notable omission and commission errors were still present when compared to reference data. Using OBT building footprints in combination with the height above nearest drainage (HAND) metric and published glacial lake outburst flood (GLOF) inundation models, we revealed that exposure to potential flooding has increased markedly over recent years. Between 2016 and 2023, building area located within 0–50 m vertical proximity to river channels increased by approximately 46%, while building area intersecting modelled GLOF extents for high-risk glacial lakes increased by 77%. Population projections carried greater uncertainty but also suggest continued growth of populations in areas of potential flood exposure, likely driven by infrastructure development and tourism, although outward migration trends to urban areas may reduce this. Overall, the population within 0–50 m HAND was projected to increase by 17,900 (6%), 2015–2030, although these population projections carry high uncertainty. The approaches demonstrated here provide a framework for monitoring exposure dynamics and offer critical baseline information to support disaster risk management and adaptation in high mountain regions. Crucially, our results highlight the importance of validation when using building footprint datasets, to avoid propagating spatial and temporal biases in derived analyses.

## Methods

### Study area

Nepal and the transboundary regions of India and China (Fig. 1) are largely influenced by the Indian Summer Monsoon, which brings the majority of precipitation between June and September and is projected to increase in intensity^1,88,89^. The occurrence of natural hazards often coincide with the monsoon season, including fatal landslides^90,91^, debris flows^3,92^, and fluvial and pluvial flooding^93^. Additionally, glacier mass loss and the formation of glacial lakes create widespread GLOF hazards in mountain catchments^50,94,95^, including the potential for transboundary floods that complicate disaster risk management strategies^79,96–98^. The transboundary study region (Fig. 1) was derived using HydroSHEDs catchments to capture glacial lakes with flow paths draining into Nepal^99^. A dataset of moraine dammed glacial lakes from Maharjan et al.^55^ was used to define glacial lake catchments. This inventory is dated 2005 ± 2 years but provides a consistent moraine-dammed lake inventory, which Veh et al.^54^ used to derive potential GLOF volumes and peak discharges for each lake. The flow path catchments from these lakes span the full extent of the mountain areas spanning the Nepal and transboundary study region. Therefore, the formation of new lakes or lake expansion would not affect our exposure assessment since their flow paths are already represented in the study domain.

### Topographic data

The 30 m ALOS Global Digital Surface Model (AW3D30) v4.1 was downloaded from JAXA to provide topographic data. The AW3D30 is the open access version of a suite of higher resolution commercially available DEMs that were derived from 2.5 m resolution ALOS-PRISM imagery collected 2006–2011^100,101^. The high native resolution often leads to improved accuracy compared to other global DEMs, with an RMSE of ~ 5 m in flat areas and up to 14 m in topographically complex areas^102–104^. The AW3D30 was hydrologically conditioned to derive a stream network using Whitebox’s *Breach Depressions Least Cost* tool, with a maximum breach distance of 2 km, and filling of remaining sinks. Breaching DEM sinks is generally preferable to filling algorithms in topographically complex catchments to avoid creating large flat artefacts behind channel blockages^105,106^.

### Building exposure

Open access building footprint datasets were downloaded for the study region (Table 3) including community digitised OpenStreetMap^107^; deep learning derived Google Open Buildings V3^48^, Google Open Buildings 2.5D Temporal (OBT)^49^, and datasets that conflate footprints from multiple sources including the OpenBuildingMap^108^ and Overture Maps buildings^109^, which for example integrates Microsoft’s Bing Maps Global ML Building Footprints^47^. A unified, nationally validated database of building footprints is not currently available for Nepal.

**Table 3:** Building footprint datasets evaluated.

Dataset	Description	Date	Source
Google Open Buildings V3	Deep learning derived building footprints derived from 0.5 m resolution satellite imagery. Includes a confidence score attribute.	Unknown. Model inferencing was carried out in May 2023 on the most up-to-date available imagery	https://sites.research.google/gr/open-buildings/
Google Open Buildings 2.5D Temporal	Deep learning derived building detection confidence raster with height estimate. Derived from Sentinel-2 imagery.	Annual timesteps 2016–2023	https://developers.google.com/earth-engine/datasets/catalog/GOOGLE_Research_open-buildings-temporal_v1
OpenBuildingMap	Conflated building footprint dataset incorporating Google Open Buildings, Global ML Building Footprints, and OpenStreetMap	Variable	https://umap.openstreetmap.de/de/map/openbuildingmap_81684#3/5.090944/−5.625000
Overture	Conflated building footprint dataset incorporating (for our study region): OpenStreetMap, Esri Community Maps, Google Open Buildings, and Global ML Building Footprints. Version: 2025-09-24.0	Variable	s3://overturemaps-us-west-2/release/2025-09-24.0
OpenStreetMap	Building footprints derived from community contributed digitised outlines.	Variable	https://www.openstreetmap.org

Exposure datasets were evaluated for catchments containing one or more glacial lakes, for a distance of 50 km downstream of the lowest lake following the flow path^36,39^. This captures the high-mountain catchments most likely to be affected by flooding, although notably GLOFs can in some cases have impacts hundreds of kilometres downstream^22^. Flow paths were derived for each individual lake (*n* = 1,361)^54,55^ using Whitebox’s *Trace Downslope Flowpaths*^110^. The flow paths were used to derive the glacial lake catchments spanning Nepal, India, and China (Fig. 1). Building footprint datasets (Table 3) were downloaded for the full study region and clipped to the glacial lake catchments to assess the total number of buildings, relative to those close to the river channel in areas of potential flood exposure (See Flood exposure). Building footprints were selected as the primary indicator of exposure because they are widely used in flood impact assessments^19,22,111,112^. When presenting comparisons, building area was reported in preference to the number of buildings, since distinguishing individual buildings is often subjective when derived from satellite data without ground-based observations. Therefore, some deep learning derived building footprint datasets can be biased towards merging adjoining or nearby buildings into a single polygon, whereas others may delineate multiple separate polygons^113^.

Building footprint datasets were cross-compared and compared to 5,994 manually digitised reference datasets spanning 2017–2025 using common accuracy assessment metrics including precision, recall, intersection-over-union, and the F1 score, which are on a 0–1 scale, where 1 is the highest accuracy^113–115^. The F1 score is a comparison metric that represents the harmonic mean of recall and precision (Eqs. 1–3). Reference building footprints were digitised using high-resolution satellite imagery in Google Earth for a series of validation sites (Supplementary Fig. 5). The precise date of the reference buildings was known from the satellite imagery at each site; however, since this was not known for the comparison datasets, the comparisons are not like-for-like, which would reduce the F1 scores. Reference footprints were used to both assess the quality and completeness of existing building footprint datasets, and to inform a threshold confidence score to derive building footprints from the Google OBT data (Supplementary Fig. 6). The OBT threshold producing the highest F1 score compared to the reference building footprints was derived across the 13 validation sites (Supplementary Fig. 5). The median threshold value (0.27) and 5th and 95th percentiles (0.18 and 0.37) were used to produce three classifications of the OBT data to define uncertainty bounds. OBT data were filtered using an elevation and snow mask, due to the presence of a small number of erroneous building detections in glacierised regions. Without access to the underlying data used to derive the OBT dataset, we could not determine the cause of these errors. Therefore, classifications over 5,500 m altitude based on the AW3D30 DEM were removed, in addition to classifications in areas of persistent snow, which was derived from a median composited normalised difference snow index^116^ (value > 0.4) derived from Sentinel-2 data spanning 2020–2023.

1 $$Precision=\frac{True\:Positive}{(True\:Positive\:+\:False\:Positive)}$$

2 $$Recall=\frac{True\:Positive}{(True\:Positive\:+\:False\:Negative)}$$

3 $$F1\:score\:=\frac{(Precision\:\times\:\:Recall)}{\left(\frac{\left(Precision\:+\:Recall\right)}{2}\right)}\:$$

### Population exposure

Population exposure was estimated using constrained WorldPop population data at 100 m resolution (2015–2030)^56^. WorldPop population estimates were generated using two census datapoints and a random forest model trained on geospatial covariates that correlate with population density. WorldPop combined this model with population projections and a built-settlement growth model to disaggregate population counts to annual 100 m grids of population. The WorldPop data were compared with Nepal census data from 2021^75^ for 140 wards (Supplementary Fig. 7). The National Population and Housing Census 2021 is the 12th national census of Nepal, conducted by National Statistics Office under Office of the Prime Minister and Council of Ministers. The census provides comprehensive demographic, social and economic information at different administrative levels including wards, which are the smallest administrative units in Nepal.

### Flood exposure

Regional scale flood hazard modelling across the Himalaya is typically carried out using ≥ 30 m global DEM products owing to the lack of higher resolution topographic data^25,65^, which only exists for local case studies since it requires field surveys or access to commercial satellite data. The 8 m High Mountain Asia (HMA) DEMs can provide improved accuracy locally^117^ but contain data gaps that restricts their use in multi-catchment flood modelling^19^. Therefore, the 30 m AW3D30 DEM was used, and in this study, the height above nearest drainage (HAND) was used to quantify relative exposure to potential flooding, without requiring a probabilistic flood return period framework^40–42^. HAND is a geomorphic indicator that is indicative of relative local flood exposure; however, dynamic elements of flooding including hydraulic constrictions, pulses, temporary damming, and seasonal water levels are not represented. HAND does not represent a flood hazard model, where event magnitudes are derived for a range of return periods. HAND was derived using Whitebox’s *Elevation Above Stream* tool applied to a hydrologically conditioned AW3D30 DEM^110^. The AW3D30 was derived from optical satellite data, and although other DEMs exists, such as the radar-derived GLO-30, studies suggest similar accuracy in mountainous topography^103,118^. HAND was derived separately for (i) glacial lake flow paths, and (ii) for glacial lake flow paths in addition to all other river channels in the study area, thereby allowing distinction between flood exposure related to glacial lakes, and non-glacial lake flow paths. The median HAND value was derived for building footprint polygons to report the area of buildings within specified elevation bands of the 50 km flow paths, therefore providing a metric that was comparable across the study area. HAND was reported over a 0–50 m range in 10 m intervals. This range was selected as a conservative measure of relative topographic exposure and proximity to valley bottoms and river corridors in steep Himalayan catchments. In these environments, extreme floods, debris flows, and GLOFs can affect areas located tens of metres above the active channel through inundation, erosion, sediment aggradation, and secondary processes such as landslide initiation^22,63^. Additionally, published scenario-based GLOF model outputs from Zhang et al. (2023)^19^ were used to quantify the change in building footprint exposure through time using OBT footprints. Zhang et al. (2023) modelled flow paths for glacial lakes with a high outburst potential, of which 285 were within our study area (Supplementary Fig. 8). These GLOF inundation extents are scenario-based using estimated drainage volume and peak discharges but do not represent the time-evolving component of GLOF hazard, which will change alongside exposure. We also merged these flow paths and applied a minimum flood depth threshold of 0.2 m to exclude negligible inundation depths and intersect with the building footprints.

## Supplementary Information

Below is the link to the electronic supplementary material.

Supplementary Material 1

## Data Availability

Supporting datasets will be downloadable from the Zenodo repository https://doi.org/10.5281/zenodo.19843432.

## References

[1] Hrudya, P. H., Varikoden, H. & Vishnu, R. A review on the Indian summer monsoon rainfall, variability and its association with ENSO and IOD. *Meteorol. Atmos. Phys.* **133**, 1–14 (2021).

[2] Pokharel, A. K. & Hallett, J. Distribution of rainfall intensity during the summer monsoon season over Kathmandu, Nepal. *Weather* **70**, 257–261 (2015).

[3] Adhikari, T. R. et al. Evaluation of post extreme floods in high mountain region: A case study of the Melamchi flood 2021 at the Koshi River Basin in Nepal. *Natural Hazards Research* **3**, 437–446 (2023).

[4] Bhambri, R. et al. Devastation in the Kedarnath (Mandakini) Valley, Garhwal Himalaya, during 16–17 June 2013: a remote sensing and ground-based assessment. *Nat. Hazards*. **80**, 1801–1822 (2016).

[5] Shrestha, F. et al. A comprehensive and version-controlled database of glacial lake outburst floods in High Mountain Asia. *Earth Syst. Sci. Data*. **15**, 3941–3961 (2023).

[6] Ioan, S., Roseo, F. & Brambilla, M. Mountain ecosystem services under a changing climate: A global perspective. *Ecosyst. Serv.* **73**, 101732 (2025).

[7] Haeberli, W. & Whiteman, C. Snow and ice-related hazards, risks, and disasters: Facing challenges of rapid change and long-term commitments. In *Snow and ice-related hazards, risks, and disasters* 1–33 (Elsevier, 2021).

[8] Haeberli, W., Schaub, Y. & Huggel, C. Increasing risks related to landslides from degrading permafrost into new lakes in de-glaciating mountain ranges. *Geomorphology* **293**, 405–417 (2017).

[9] Stanley, T. A., Soobitsky, R. B., Amatya, P. M. & Kirschbaum, D. B. Landslide hazard is projected to increase across High Mountain Asia. *Earths Future* **12**, e2023EF004325 (2024).

[10] Shugar, D. H. et al. A massive rock and ice avalanche caused the 2021 disaster at Chamoli, Indian Himalaya. *Science*10.1126/science.abh4455 (2021). 34112725

[11] Kumar, A., Mal, S., Schickhoff, U. & Dimri, A. P. Basin-scale spatio-temporal development of glacial lakes in the Hindukush-Karakoram-Himalayas. *Glob Planet. Change*. **245**, 104656 (2025).

[12] Shugar, D. H. et al. Rapid worldwide growth of glacial lakes since 1990. *Nat. Clim. Change* **10**, 939–945 (2020).

[13] Zhang, T., Wang, W. & An, B. Heterogeneous changes in global glacial lakes under coupled climate warming and glacier thinning. *Commun. Earth Environ.* **5**, 374 (2024).

[14] Harrison, S. et al. Climate change and the global pattern of moraine-dammed glacial lake outburst floods. *Cryosphere ***12**, 1195–1209 (2018).

[15] Veh, G. et al. Trends, Breaks, and Biases in the Frequency of Reported Glacier Lake Outburst Floods. *Earths Future ***10**, 1–14 (2022).

[16] Zhang, T. et al. High frequency of moraine-dammed lake outburst floods driven by global warming. *Nat. Commun.* **16**11173. (2025). 10.1038/s41467-025-67650-3 PMC12711897 41407701

[17] GAPHAZ. Assessment of Glacier and Permafrost Hazards in Mountain Regions – Technical Guidance Document. Prepared by Allen, S., Frey, H., Huggel, C. et al. Standing Group on Glacier and Permafrost Hazards in Mountains (GAPHAZ) of the International Association of Cryospheric Sciences (IACS) and the International Permafrost Association (IPA). Zurich, Switzerland / Lima, Peru, 72 pp et al. (2017).

[18] Carrivick, J. L. & Tweed, F. S. A global assessment of the societal impacts of glacier outburst floods. *Glob Planet. Change*. **144**, 1–16 (2016).

[19] Zhang, T., Wang, W., An, B. & Wei, L. Enhanced glacial lake activity threatens numerous communities and infrastructure in the Third Pole. *Nat. Commun. ***14**, 8250 (2023). 10.1038/s41467-023-44123-z PMC10716169 38086866

[20] Chen, H., Liang, Q., Zhao, J. & Maharjan, S. B. Assessing national exposure to and impact of glacial lake outburst floods considering uncertainty under data sparsity. *Hydrol. Earth Syst. Sci. ***29**, 733–752 (2025).

[21] Bai, Y. et al. Flood complexity and rising exposure risk in High Mountain Asia under climate change. *Sci. Bull. ***70**, 1601–1604 (2025). 10.1016/j.scib.2025.01.055 39988524

[22] Sattar, A. et al. The Sikkim flood of October 2023: Drivers, causes and impacts of a multihazard cascade. *Science ***387**, eads2659 (2025). 10.1126/science.ads2659 39883788

[23] Sattar, A., Emmer, A., Lhazom, T., Rai, S. K. & Azam, M. F. Flood risk from small mountain lakes. *Commun. Earth Environ.* **6**, 785 (2025).

[24] IPCC. IPCC SR Ocean and Cryosphere. Chapter 2: High Mountain Areas. (2019).

[25] Pantha, R. & Sharma, B. R. Population size, growth and distribution. *Popul. Monogr. Nepal. ***1**, 37–86 (2003).

[26] Wang, N. et al. Flood fatalities and displacement influence human migration in floodplains of developing countries. *Commun. Earth Environ.* **6**, 319 (2025).

[27] Jones, T. E., Apollo, M. & Bui, H. T. Mountainous Protected Areas & Nature-Based Tourism in Asia. In *Nature-Based Tourism in Asia’s Mountainous Protected Areas: A Trans-regional Review of Peaks and Parks* (eds Jones, T. E. et al.) 3–25 (Springer International Publishing, 2021). 10.1007/978-3-030-76833-1_1.

[28] Nyaupane, G. P. & Chhetri, N. Vulnerability to climate change of nature-based tourism in the Nepalese Himalayas. *Tourism Geogr.* **11**, 95–119 (2009).

[29] Pawson, I. G., Stanford, D. D., Adams, V. A. & Nurbu, M. Growth of tourism in Nepal’s Everest region: Impact on the physical environment and structure of human settlements. *Mt. Res. Dev.* **4**, 237–246 (1984).

[30] Watson, C. S. & King, O. Everest’s thinning glaciers: implications for tourism and mountaineering. *Geol. Today*. **34**, 18–25 (2018).

[31] Li, D. et al. High mountain Asia hydropower systems threatened by climate-driven landscape instability. *Nat. Geosci.* **15**, 520–530 (2022).

[32] Schwanghart, W., Worni, R., Huggel, C., Stoffel, M. & Korup, O. Uncertainty in the Himalayan energy–water nexus: estimating regional exposure to glacial lake outburst floods. *Environ. Res. Lett. ***11**, 1–9 (2016).

[33] Emmer, A. et al. Progress and challenges in glacial lake outburst flood research (2017–2021): a research community perspective. *Nat. Hazards Earth Syst. Sci. ***22**, 3041–3061 (2022).

[34] Collins, T. W., Grineski, S. E. & Chakraborty, J. Environmental injustice and flood risk: A conceptual model and case comparison of metropolitan Miami and Houston, USA. *Reg. Environ. Change* **18**, 311–323 (2018). 10.1007/s10113-017-1121-9 PMC5849275 29551952

[35] Blaikie, P., Cannon, T., Davis, I. & Wisner, B. *At Risk: Natural Hazards, People’s Vulnerability and Disasters* (Routledge, 2014).

[36] Dubey, S. et al. Mass Movement Hazard and Exposure in the Himalaya. 11, e2022EF003253 (2023).

[37] Kincey, M. E. et al. National-scale rainfall-triggered landslide susceptibility and exposure in Nepal. *Earths Future* **12**, e2023EF004102 (2024).

[38] Tellman, B. et al. Satellite imaging reveals increased proportion of population exposed to floods. *Nature* **596**, 80–86 (2021). 10.1038/s41586-021-03695-w 34349288

[39] Taylor, C., Robinson, T. R., Dunning, S., Carr, J. R. & Westoby, M. Glacial lake outburst floods threaten millions globally. *Nat. Commun.* **14**487. (2023). 10.1038/s41467-023-36033-x PMC9905510 36750561

[40] McGrath, H., Bourgon, J.-F., Proulx-Bourque, J.-S., Nastev, M. & Abo El Ezz, A. A comparison of simplified conceptual models for rapid web-based flood inundation mapping. *Nat. Hazards* **93**, 905–920 (2018).

[41] Nobre, A. D. et al. Height above the nearest drainage – a hydrologically relevant new terrain model. *J. Hydrol.* **404**, 13–29 (2011).

[42] Rennó, C. D. et al. HAND, a new terrain descriptor using SRTM-DEM: Mapping terra-firme rainforest environments in Amazonia. *Remote Sens. Environ.* **112**, 3469–3481 (2008).

[43] Watson, C. S., Gyawali, J., Creed, M. & Elliott, J. R. City-scale high-resolution flood models and the role of topographic data: a case study of Kathmandu, Nepal. *Geocarto Int. ***39**, 2387073 (2024).

[44] Hecht, R. et al. Measuring Completeness of Building Footprints in OpenStreetMap over Space and Time. *ISPRS Int. J. Geo-Inf*. **2**, 1066–1091 (2013).

[45] Herfort, B., Lautenbach, S., Porto de Albuquerque, J., Anderson, J. & Zipf, A. A spatio-temporal analysis investigating completeness and inequalities of global urban building data in OpenStreetMap. *Nat. Commun. ***14**, 3985 (2023). 10.1038/s41467-023-39698-6 PMC10326063 37414776

[46] Watson, C. S. & Elliott, J. R. Narrowing the gap for city building height predictions. *Sci. Rep. ***15**, 29913 (2025). 10.1038/s41598-025-15929-2 PMC12354800 40813610

[47] Microsoft *Global ML Building Footprints. [Online]. Accessed 21st May* (2024). Available from: https://Github.Com/Microsoft/*GlobalMLBuildingFootprints*. (2024).

[48] Sirko, W. et al. Continental-Scale Building Detection from High Resolution Satellite Imagery. *ArXiv* abs/2107.12283, (2021).

[49] Sirko, W. et al. High-Resolution Building and Road Detection from Sentinel-2. Preprint at (2024). 10.48550/arXiv.2310.11622

[50] Rounce, D., Watson, C. & McKinney, D. Identification of hazard and risk for glacial lakes in the Nepal Himalaya using satellite imagery from 2000–2015. *Remote Sens.* 10.3390/rs9070654 (2017).

[51] Esch, T. et al. World Settlement Footprint 3D-A first three-dimensional survey of the global building stock. *Remote Sens. Environ* **270**, 1–15 (2022).

[52] Ressl, C. & Pfeifer, N. Evaluation of the elevation model influence on the orthorectification of Sentinel-2 satellite images over Austria. *Eur. J. Remote Sens.* **51**, 693–709 (2018).

[53] Wang, H. & Ellis, E. C. Image misregistration error in change measurements. *Photogramm Eng. Remote Sens.* **71**, 1037–1044 (2005).

[54] Veh, G., Korup, O. & Walz, A. Hazard from Himalayan glacier lake outburst floods. 201914898 doi:10.1073/pnas.1914898117%J Proceedings of the National Academy of Sciences. (2019). 10.1073/pnas.1914898117 PMC6969545 31888996

[55] Maharjan, S. B. et al. The status of glacial lakes in the Hindu Kush Himalaya-ICIMOD Research Report 2018/1 (2018). https://www.cabidigitallibrary.org/doi/full/10.5555/20183233484 (2018).

[56] Bondarenko, M. et al. Constrained estimates of 2015–2030 total number of people per grid square at a resolution of 3 arc (approximately 100m at the equator) R2025A version v1. *Global Demographic Data Project - Funded Bill Melinda Gates Foundation (INV-045237) WorldPop - School Geogr. Environ. Sci. Univ. Southampt.* 10.5258/SOTON/WP00839 (2025).

[57] Wickham, H. Data Analysis. In *ggplot2* 189–201 (Springer International Publishing, 2016). 10.1007/978-3-319-24277-4_9.

[58] Kirschbaum, D., Kapnick, S. B., Stanley, T. & Pascale, S. Changes in Extreme Precipitation and Landslides Over High Mountain Asia. *Geophys. Res. Lett.* **47**, e2019GL085347 (2020).

[59] Karki, R., Hasson, S., ul, Schickhoff, U., Scholten, T. & Böhner, J. Rising Precipitation Extremes across Nepal. *Climate* **5**, 4 (2017).

[60] Ullah, T., Lautenbach, S., Herfort, B., Reinmuth, M. & Schorlemmer, D. Assessing Completeness of OpenStreetMap Building Footprints Using MapSwipe. *ISPRS Int. J. Geo-Inf*. **12**, 143 (2023).

[61] Dabove, P., Daud, M. & Olivotto, L. Revolutionizing urban mapping: Deep learning and data fusion strategies for accurate building footprint segmentation. *Sci. Rep.* **14**13510. (2024). 10.1038/s41598-024-64231-0 PMC11169381 38866920

[62] Maharjan, S. B. et al. The Melamchi flood disaster: Cascading hazard and the need for multihazard risk management. *Kathmandu Nepal. ICIMOD.* 1–21. https://www.icimod.org/article/the-melamchi-flood-disaster/ (2021).

[63] Chen, C.-M. et al. Erosional cascade during the 2021 Melamchi flood. *Nat. Geosci.* **18**, 32–36 (2025).

[64] Byers, A. C. et al. The Changing Ecology of a High Himalayan Valley: Challenges to the Sustainable Development of the Kanchenjunga Conservation Area, Eastern Nepal. *Sustainability* **16**, 2434 (2024).

[65] Sharma, R. H. & Awal, R. Hydropower development in Nepal. *Renew. Sustain. Energy Rev.* **21**, 684–693 (2013).

[66] Rimal, B., Baral, H., Stork, N. E., Paudyal, K. & Rijal, S. Growing city and rapid land use transition: Assessing multiple hazards and risks in the Pokhara Valley, Nepal. *Land* **4**, 957–978 (2015).

[67] Government of Nepal. Nepal National Disaster Risk Reduction Policy. (2018). http://drrportal.gov.np/uploads/document/1476.pdf

[68] Gurung, N., Arnaud-Fassetta, G., Fort, M., Bell, R. & Sherchan, B. Construction of road bridges without consideration of geo-hazards and river-flow dynamics–a case study from Ghatte Khola, Myagdi, Nepal. *Géomorphologie Relief Process. Environ.* **26**, 195–215 (2020).

[69] Nepal, S., Flügel, W.-A. & Shrestha, A. B. Upstream-downstream linkages of hydrological processes in the Himalayan region. *Ecol. Process.* **3**, 19 (2014).

[70] Tandon, A. & Menon, A. Flood risk study of Himalayan valley highlights unregulated tourism as a cause. *Mongabay-India* (2024). https://india.mongabay.com/2024/07/flood-risk-study-of-himalayan-valley-highlights-unregulated-tourism-as-a-cause/

[71] Fischer, M. et al. Rare flood scenarios for a rapidly growing high-mountain city: Pokhara, Nepal. *Nat. Hazards Earth Syst. Sci.* **22**, 3105–3123 (2022).

[72] Gurung, P. & Pokhrel, R. Climate Change Vulnerability Assessment in Informal Urban Settlements. *J. Eng. Sci.* **4**, 109–120 (2025).

[73] Kumar, A. Impact of building regulations on Indian hill towns. *HBRC J.* **12**, 316–326 (2016).

[74] Bergmann, C., Gerwin, M., Nüsser, M. & Sax, W. S. Living in a high mountain border region: The case of the ‘Bhotiyas’ of the Indo-Chinese border region. *J. Mt. Sci.* **5**, 209–217 (2008).

[75] National Statistics Office, Government of Nepal. National Population and Housing Census (NHPC). (2021). https://censusresults.nsonepal.gov.np/

[76] Láng-Ritter, J., Keskinen, M. & Tenkanen, H. Global gridded population datasets systematically underrepresent rural population. *Nat. Commun.* **16**, 2170 (2025). 10.1038/s41467-025-56906-7 PMC11920052 40102392

[77] Bhatt, P. & Joshi, K. R. Hydropower development in Nepal: Status, opportunities and challenges. *J. UTEC Eng. Manag*. **2**, 125–136 (2024).

[78] Gouli, M. R. et al. Quantitative assessment of the GLOF risk along China-Nepal transboundary basins by integrating remote sensing, machine learning, and hydrodynamic model. *Int. J. Disaster Risk Reduct.* **118**, 105231 (2025).

[79] Khadka, N. et al. Glacial lake outburst floods threaten China-Nepal connectivity: Synergistic study of remote sensing, GIS and hydrodynamic modeling with regional implications. *Sci. Total Environ.* **948**, 174701 (2024). 10.1016/j.scitotenv.2024.174701 38997012

[80] Nepal, S. Tourism and remote mountain settlements: Spatial and temporal development of tourist infrastructure in the Mt Everest Region, Nepal. *Tourism Geogr.* **7**, 205–227 (2005).

[81] Lamichhane, K. et al. Unraveling the causes and impacts of increasing flood disasters in the kathmandu valley: Lessons from the unprecedented September 2024 floods. *Nat. Hazards Res.* **5**, 875–897 (2025).

[82] Khanal, N. R. Floods in mountain watershed: a case of Madi River, central Nepal. *J. Hydrol. Meteorol.* **1**, 67–73 (2004).

[83] Regmi, D., Bhatta, K. & Dahal, S. Mountain tourism and hazards: A proposal for a new direction of mountain tourism in Nepal. *Glob Environ. Res.* **27**, 133–138 (2023).

[84] Childs, G., Craig, S., Beall, C. M. & Basnyat, B. Depopulating the Himalayan Highlands: Education and outmigration from ethnically Tibetan communities of Nepal. *Mt. Res. Dev.* **34**, 85–94 (2014).

[85] Kc, B., Wang, T. & Gentle, P. Internal Migration and Land Use and Land Cover Changes in the Middle Mountains of Nepal. *Mt. Res. Dev.* **37**, 446–455 (2017).

[86] Watanabe, T. & Rothacher, D. The 1994 Lugge Tsho Glacial Lake Outburst Flood, Bhutan Himalaya. *Mt. Res. Dev.* **16**, 77–81 (1996).

[87] Cai, B., Shao, Z., Huang, X., Zhou, X. & Fang, S. Deep learning-based building height mapping using Sentinel-1 and Sentinel-2 data. *Int. J. Appl. Earth Obs Geoinf.* **122**, 103399 (2023).

[88] Bookhagen, B. & Burbank, D. W. Topography, relief, and TRMM-derived rainfall variations along the Himalaya. *Geophys. Res. Lett.* **33**, 1–5 (2006).

[89] Kitoh, A. The Asian monsoon and its future change in climate models: A review. *J. Meteorol. Soc. Jpn Ser. II*. **95**, 7–33 (2017).

[90] Dahal, R. K. & Hasegawa, S. Representative rainfall thresholds for landslides in the Nepal Himalaya. *Geomorphology* **100**, 429–443 (2008).

[91] Petley, D. N. et al. Trends in landslide occurrence in Nepal. *Nat. Hazards*. **43**, 23–44 (2007).

[92] Adhikari, D. P. & Koshimizu, S. Debris flow disaster at Larcha, upper Bhotekoshi Valley, central Nepal. *Isl Arc*. **14**, 410–423 (2005).

[93] Pangali Sharma, T. P. et al. Review of flood disaster studies in Nepal: A remote sensing perspective. *Int. J. Disaster Risk Reduct.* **34**, 18–27 (2019).

[94] Bajracharya, S. R. & Mool, P. Glaciers, glacial lakes and glacial lake outburst floods in the Mount Everest region, Nepal. *Ann. Glaciol*. **50**, 81–86 (2009).

[95] Khadka, N., Zhang, G. & Thakuri, S. Glacial lakes in the Nepal Himalaya: Inventory and decadal dynamics (1977–2017). *Remote Sens.* **10**, 1913 (2018).

[96] Allen, S. K. et al. Glacial lake outburst flood hazard under current and future conditions: Worst-case scenarios in a transboundary Himalayan basin. *Nat. Hazards Earth Syst. Sci.* **22**, 3765–3785 (2022).

[97] Khanal, N. R. et al. A comprehensive approach and methods for glacial lake outburst flood risk assessment, with examples from Nepal and the transboundary area. *Int. J. Water Resour. Dev.* **31**, 219–237 (2015).

[98] Sattar, A., Haritashya, U. K., Kargel, J. S. & Karki, A. Transition of a small Himalayan glacier lake outburst flood to a giant transborder flood and debris flow. *Sci. Rep.* **12**, 12421 (2022). 10.1038/s41598-022-16337-6 PMC9300610 35858949

[99] Lehner, B., Verdin, K. & Jarvis, A. New global hydrography derived from spaceborne elevation data. *Eos, Trans. Am. Geophys. Union* **89**, 93–94 (2008).

[100] Tadono, T. et al. Precise global DEM generation by ALOS PRISM. *ISPRS Ann. Photogramm Remote Sens. Spat. Inf. Sci.* **2**, 71 (2014).

[101] JAXA. ALOS World 3D – 30 m (AW3D30), Version 4.1, Earth Observation Research Center (EORC). Earth Engine Data Catalog. (2024).

[102] Li, H. & Zhao, J. Evaluation of the Newly Released Worldwide AW3D30 DEM Over Typical Landforms of China Using Two Global DEMs and ICESat/GLAS Data. *IEEE J. Sel. Top. Appl. Earth Obs Remote Sens.* **11**, 4430–4440 (2018).

[103] Trevisani, S., Skrypitsyna, T. N. & Florinsky, I. V. Global digital elevation models for terrain morphology analysis in mountain environments: Insights on Copernicus GLO-30 and ALOS AW3D30 for a large Alpine area. *Environ. Earth Sci.* **82**, 198 (2023).

[104] Uuemaa, E., Ahi, S., Montibeller, B., Muru, M. & Kmoch, A. Vertical accuracy of freely available global digital elevation models (ASTER, AW3D30, MERIT, TanDEM-X, SRTM, and NASADEM). *Remote Sens.* **12**, 3482 (2020).

[105] Lidberg, W., Nilsson, M., Lundmark, T. & Ågren, A. M. Evaluating preprocessing methods of digital elevation models for hydrological modelling. *Hydrol. Process.* **31**, 4660–4668 (2017).

[106] Lindsay, J. B. Efficient hybrid breaching-filling sink removal methods for flow path enforcement in digital elevation models. *Hydrol. Process.* **30**, 846–857 (2016).

[107] OpenStreetMap contributors. OpenStreetMap. [online]. Accessed 09. October (2025). Available from: https://www.openstreetmap.org. (2025).

[108] Oostwegel, L. J. N., Schorlemmer, D. & Guéguen, P. From footprints to functions: A comprehensive global and semantic building footprint dataset. *Sci. Data* **12**, 1699 (2025). 10.1038/s41597-025-06132-z PMC12559435 41145547

[109] Overture Maps Foundation. overturemaps.org [online]. Accessed 23rd. (2025).October (2025).

[110] Lindsay, J. B. Whitebox GAT: A case study in geomorphometric analysis. *Comput. Geosci.* **95**, 75–84 (2016).

[111] Rinzin, S., Dunning, S., Carr, R. J., Sattar, A. & Mergili, M. Exploring implications of input parameter uncertainties in glacial lake outburst flood (GLOF) modelling results using the modelling code r.avaflow. *Nat. Hazards Earth Syst. Sci.* **25**, 1841–1864 (2025).

[112] Zheng, G. et al. Increasing risk of glacial lake outburst floods from future Third Pole deglaciation. *Nat. Clim. Change*. **11**, 411–417 (2021).

[113] Dabove, P., Daud, M. & Olivotto, L. Revolutionizing urban mapping: Deep learning and data fusion strategies for accurate building footprint segmentation. *Sci. Rep.* **14**, 13510 (2024). 10.1038/s41598-024-64231-0 PMC11169381 38866920

[114] Li, W. et al. Semantic segmentation-based building footprint extraction using very high-resolution satellite images and multi-source GIS data. *Remote Sens.* 1–19. 10.3390/rs11040403 (2019).

[115] Schuegraf, P., Bittner, K., Schuegraf, P. & Bittner, K. Automatic building footprint extraction from multi-resolution remote sensing images using a hybrid FCN. *ISPRS Int. J. Geo-Inf.* 1–16. 10.3390/ijgi8040191 (2019).

[116] Hall, D. K., Riggs, G. A. & Salomonson, V. V. Development of methods for mapping global snow cover using moderate resolution imaging spectroradiometer data. *Remote Sens. Environ.* **54**, 127–140 (1995).

[117] Shean, D. July High Mountain Asia 8-meter DEM Mosaics Derived from Optical Imagery, Version 1. Boulder, Colorado USA. NASA National Snow and Ice Data Center Distributed Active Archive Center. https://doi.org/10.5067/KXOVQ9L172S2. Accessed 19 (2017) (2018). . Accessed 19 July 2018.

[118] Pandya, D., Rana, V. K. & Suryanarayana, T. M. V. Inter-comparison and assessment of digital elevation models for hydrological applications in the Upper Mahi River Basin. *Appl. Geomat.* **16**, 191–214 (2024).

