# Supplementary material for: Increasing exposure to potential flooding in high-mountain catchments
Source: Sci Rep. 2026 Oct 8;16:29730. doi: 10.1038/s41598-026-70268-0 (PMC13649566; doi:10.1038/s41598-026-70268-0)
Supplement: Supplementary file 1 [file 41598_2026_70268_MOESM1_ESM.docx]

**Supplementary information: Increasing exposure to potential flooding in high-mountain catchments**

C. Scott Watson^1^, Lauren D. Rawlins^1,^ Mohan B. Chand^2^, Rakesh Bhambri^3^, Nitesh Khadka^4^, Milan Shrestha^5^, Deepak KC^6^, Daniel Routledge^1^, and Grace Middleditch^1^

^1^School of Geography and water@leeds, University of Leeds, Leeds LS2 9JT, United Kingdom.

^2^Department of Environmental Science and Engineering, Himalayan Cryosphere, Climate and Disaster Research Centre (HiCCRDC), School of Science, Kathmandu University, Dhulikhel, Nepal.

^3^Department of Geography, University of Delhi, India.

^4^Centre for Water Resources Studies (CWRS), Institute of Engineering, Tribhuvan University, Nepal.

^5^School of Sustainability, Arizona State University, USA.

^6^United Nations Development Programme Nepal, Kathmandu, Nepal.

**Supplementary Fig. 1.** Building dataset area distributions and catchment hypsometry (grey shaded area) for the glacial lake catchments. (a-c) Elevation hypsometry and building area distribution by dataset and country. (d-f) Building area distribution with increasing HAND. The HAND drainage network represents downstream flow paths from glacial lakes.

**Supplementary Fig. 2.** (a) Violin plot showing the accuracy assessment of the building footprint datasets for 32 reference datasets that were digitised across 13 validation sites. Median values are shown on the plot. (b) Total magnitude of the registration shift applied to each building dataset to optimise the alignment with the reference dataset.

**Supplementary Fig. 3.** Cumulative OBT building area with distance downstream from glacial lakes, coloured by year of the dataset.

**Supplementary Fig. 4.** Change in population through time from WorldPop data in the study catchments, for populations in glacial lake flow paths. (a–d) Population summed by HAND bin from 0 to 50 m above the river channel. (e–h) Population summed into a single HAND bin of 0 to 50 m above the river channel. (i–l) Total population in the lake catchments.

**Supplementary Fig. 5.** Location of the 13 validation sites that were selected to derive building footprint reference data using high resolution satellite imagery in Google Earth Pro. Figure created in QGIS 3.34.14.

**Supplementary Fig. 6:** OBT data for Lamachaur. (a) Variation in the F1 score and optimum (black marker) with increasing OBT threshold, when compared to the reference datasets. (b) Distribution of OBT presence values for cells intersecting the reference building footprints, and those not (non-reference).

**Supplementary Fig. 7.** Location of the 140 wards used to compare 2021 Nepal census data with WorldPop data. Population difference between the two datasets is shown aggregated to ward boundaries. Figure created in QGIS 3.34.14.

**Supplementary Fig. 8.** Location of the 285 flood model outputs of Zhang et al. (2023)^1^ that were used to quantify inundated buildings in the OBT dataset. Figure created in QGIS 3.34.14.

**References**

1.	Zhang, T., Wang, W., An, B. & Wei, L. Enhanced glacial lake activity threatens numerous communities and infrastructure in the Third Pole. *Nat. Commun.* **14**, 8250 (2023).
